# Epidural electrocorticography of phantom hand movement following long-term upper-limb amputation

**DOI:** 10.3389/fnhum.2014.00285

**Published:** 2014-05-06

**Authors:** Alireza Gharabaghi, Georgios Naros, Armin Walter, Alexander Roth, Martin Bogdan, Wolfgang Rosenstiel, Carsten Mehring, Niels Birbaumer

**Affiliations:** ^1^Division of Functional and Restorative Neurosurgery and Division of Translational Neurosurgery, Department of Neurosurgery, Eberhard Karls University of TübingenTübingen, Germany; ^2^Neuroprosthetics Research Group, Werner Reichardt Centre for Integrative Neuroscience, Eberhard Karls University of TübingenTübingen, Germany; ^3^Department of Computer Engineering, Wilhelm-Schickard Institute for Computer Science, Eberhard Karls University of TübingenTübingen, Germany; ^4^Department of Computer Engineering, University of LeipzigLeipzig, Germany; ^5^Institute for Biology III, Albert-Ludwigs-UniversityFreiburg im Breisgau, Germany; ^6^Institute of Medical Psychology and Behavioural Neurobiology, Eberhard Karls University of TübingenTübingen, Germany

**Keywords:** electrocorticography, epidural implant, neural prosthesis, brain–computer interface, brain–machine interface, closed-loop control, amputee, neurofeedback

## Abstract

**Introduction:** Prostheses for upper-limb amputees are currently controlled by either myoelectric or peripheral neural signals. Performance and dexterity of these devices is still limited, particularly when it comes to controlling hand function. Movement-related brain activity might serve as a complementary bio-signal for motor control of hand prosthesis.

**Methods:** We introduced a methodology to implant a cortical interface without direct exposure of the brain surface in an upper-limb amputee. This bi-directional interface enabled us to explore the cortical physiology following long-term transhumeral amputation. In addition, we investigated neurofeedback of electrocorticographic brain activity related to the patient’s motor imagery to open his missing hand, i.e., phantom hand movement, for real-time control of a virtual hand prosthesis.

**Results:** Both event-related brain activity and cortical stimulation revealed mutually overlapping cortical representations of the phantom hand. Phantom hand movements could be robustly classified and the patient required only three training sessions to gain reliable control of the virtual hand prosthesis in an online closed-loop paradigm that discriminated between hand opening and rest.

**Conclusion:** Epidural implants may constitute a powerful and safe alternative communication pathway between the brain and external devices for upper-limb amputees, thereby facilitating the integrated use of different signal sources for more intuitive and specific control of multi-functional devices in clinical use.

## INTRODUCTION

Multi-functional prostheses for upper-limb amputees are usually controlled by electromyogram (EMG) signals of the remaining muscles. However, since present approaches afford the user only limited dexterity and intuitive control of these devices, particularly when patients are affected by higher levels of limb amputation (e.g., transhumeral amputation), they often meet with rather low acceptance in clinical practice (Davidson, 2002). In research settings, pattern-recognition approaches to myoelectric control and shared control strategies have been evaluated to overcome some of the current limitations (Cipriani et al., 2008; Scheme and Englehart, 2011). In addition to advanced decoding algorithms and control paradigms, several different surgical approaches have been explored to physiologically establish more appropriate control sites for upper-limb prostheses: implanted EMG sensors were evaluated to improve recording robustness and to provide simultaneous control of multiple degrees of freedom (Baker et al., 2010). Also, the surgical transfer of brachial nerves to new muscle sites was performed in patients with proximal amputations to gain access to appropriate neural information in the absence of physiologically meaningful EMG signals (Kuiken et al., 2009). In a further approach, implantable electrodes connected with peripheral nerves were explored as interfaces for bi-directional information flow to provide sensory feedback together with motor control signals (Rossini et al., 2010).

The common denominator between these different approaches is that they all acquired control signals from the peripheral neural system close to the amputation site, thereby being potentially affected by the act of wearing the prosthesis and by possible variability with regard to electrode position, force, limb position, and transient EMG changes (Scheme and Englehart, 2011).

Control signals from the central neural system recorded directly from the brain may overcome some of the present limitations of peripheral control sites. Movement-related brain activity might serve as a complementary bio-signal for intuitive motor control of hand prosthesis. Such brain–computer or brain–machine interfaces (BCI/BMI) for prosthetic control have been investigated in able-bodied individuals using both non-invasive approaches with surface electroencephalography (EEG) recordings (Lauer et al., 1999; McFarland and Wolpaw, 2008a; Muller-Putz and Pfurtscheller, 2008) and invasive approaches with implanted electrocorticographic (ECoG) recordings in epilepsy patients undergoing diagnostic recordings for seizure localization (Chestek et al., 2013). Moreover, ECoG recordings in able-bodied individuals were used for decoding natural grasp types and individual finger movements (Kubanek et al., 2009; Miller et al., 2009; Pistohl et al., 2011).

Experience with brain interfaces and prosthetic control in patients with severe motor deficits such as tetraplegia, stroke or amputation is, on the other hand, fairly limited (Pfurtscheller et al., 2000; Yanagisawa et al., 2011, 2012; Hochberg et al., 2012; Collinger et al., 2013; Wang et al., 2013).

Non-invasive BCI approaches using EEG are characterized by low spatial resolution, a low signal-to-noise-ratio due to signal attenuation caused by the skull, possible contamination by muscle artifacts and external electrical activity, and a comparatively long period of training to gain real-time control of devices (Leuthardt et al., 2009). However, by virtue of their proximity to the neural signal source, implantable BCI approaches may be able to overcome these limitations. Remarkably, all current implantable BCIs in patients with motor deficits are in direct contact with the brain via *subdural* grids (Yanagisawa et al., 2011, 2012; Wang et al., 2013) or even penetrate the tissue with *intracortical* microelectrodes (Hochberg et al., 2012; Collinger et al., 2013), thus bearing additional risks with regard to safety and stability in long-term application.

Implantable but less invasive approaches harnessing the epidural space might therefore help to improve the risk-benefit ratio and provide novel tools in this patient group. Additionally, implantable approaches have not yet been explored following long-term upper-limb amputation and cortical reorganization may limit the detection of natural movement-related brain signals.

Thus, we introduced a methodology to implant a cortical interface without direct exposure of the brain surface in an upper-limb amputee. This involved using a closed-loop set-up based on an *epidural* implant that provided real-time feedback of motor-related electrocorticographic brain activity to operate a virtual hand prosthesis following *long-term* high-level limb deficiency.

## METHODS

### PATIENT

The 63-year-old, male patient had suffered a motor-cycle accident with complete right-sided cervical nerve root avulsion and transhumeral amputation of his right upper-limb 26 years before admission to our institution. Since the accident, the patient has suffered from intractable pain despite multiple medical and surgical interventions such as deep brain stimulation of the left thalamus and the dorsal root entry zone (DREZ) procedure to treat the pain.

As a candidate for long-term cortical stimulation to reduce his chronic pain, the patient underwent implantation of an electrode array covering the left sensorimotor cortex to determine treatment response and optimal stimulation sites for maximum pain reduction. Indication for implantation, array location and duration of implantation were determined solely by clinical criteria. The patient also gave informed consent to a study (including publication of data and photographs) investigating a BCI approach for virtual hand prosthesis control during this time, which was conducted in accordance with the declaration of Helsinki and the guidelines of the local ethics committee. The results of the pain evaluation study do not constitute part of the present report.

### IMPLANTATION

**FIGURE 1:**
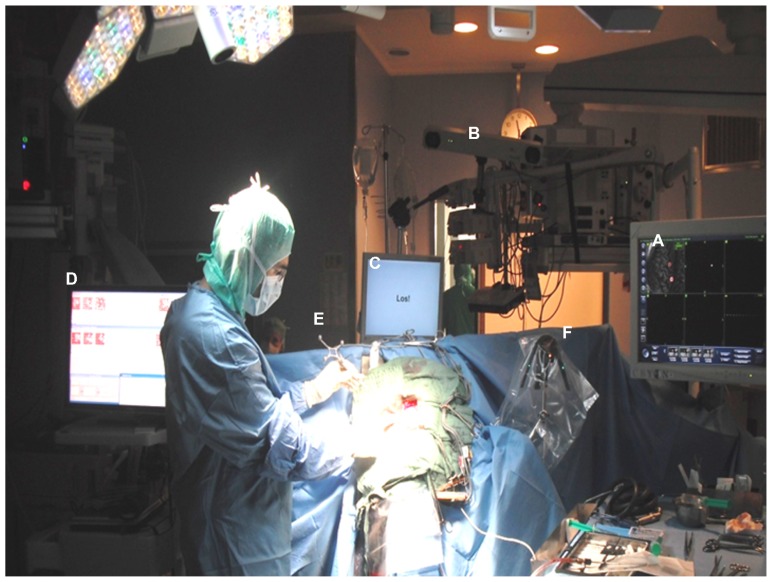
**Electrode grid implantation during awake surgery without direct exposure of the brain surface:** craniotomy and initial epidural grid placement were performed with a neuronavigational approach based on preoperative magnetic resonance images **(A)** and intraoperative optical image-guidance **(B).** Grid positioning was refined applying online ECoG mapping **(C)** during cued phantom hand movements with auditory and visual cues presented via a monitor placed at patient’s eye level **(D)**. Optimal orientation of the implant in the epidural space was achieved by tracking electrode positions with strongest task-related ECoG activity with a navigated pointer **(E)** in relation to a reference frame **(F)** and adjusting the center of the electrode grid accordingly.

The electrode grid was implanted during awake surgery without direct exposure of the brain surface. Craniotomy and initial epidural grid placement were performed with an image-guidance approach described earlier (Gharabaghi et al., 2005). For refinement of grid positioning, we implemented a novel mapping methodology of localizing the cortical representation of phantom hand movements intraoperatively during awake surgery (**Figure 1**).

**FIGURE 2:**
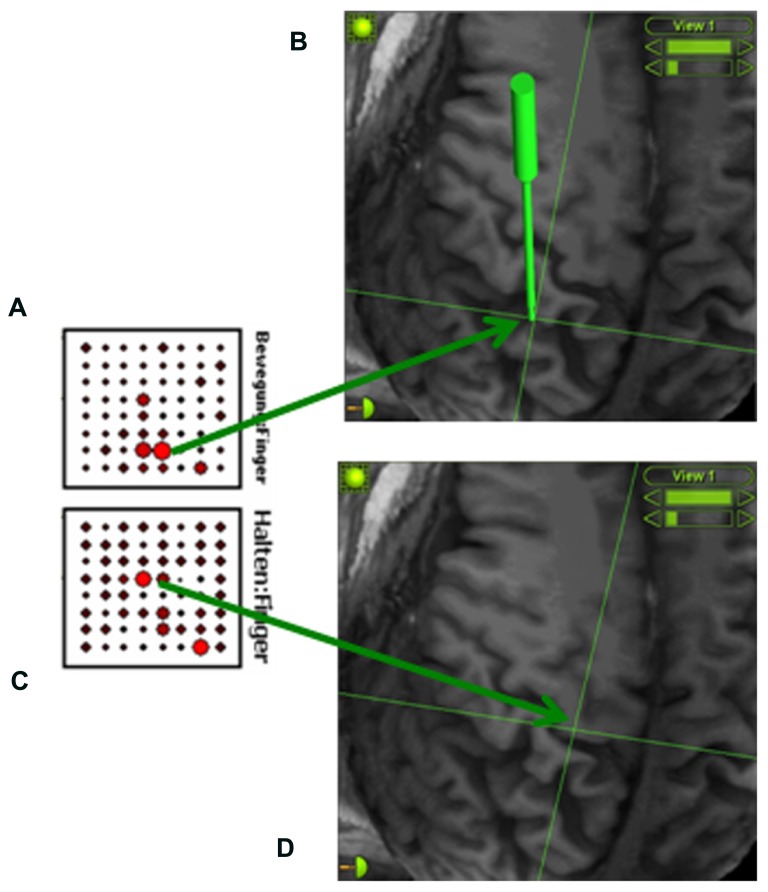
**Online detection of cortical areas related to phantom hand movement using the SIGFRIED module within the BCI2000 framework** (Schalk et al., 2008a, b). This procedure creates a statistical model of baseline brain activity and detects significant deviations from this baseline in real-time during the task. During each trial, z-scores between the distributions of negative log likelihood values for the task and interleaved rest periods are calculated, resulting in one continuously updated z-score for each electrode location and task. These z-scores are shown on a topographical display for each task (**A**: finger extension; **C**: holding finger extension) as a circle at each electrode location whose diameter is proportional to the z-scores at that location, thus enabling real-time assessment of cortical changes (Schalk et al., 2008a, b). An image-guidance approach with a navigated pointer (green) allows for relating the electrodes with the strongest activity to the underlying cortical anatomy (**B**: somatosensory cortex; **D**: primary motor cortex) without exposing the brain surface.

We applied a procedure known as signal modeling for real-time identification and event detection (SIGFRIED) within the BCI2000 framework (Schalk et al., 2004) which has recently been explored for online motor and speech mapping of epilepsy and tumor patients (Brunner et al., 2009; Roland et al., 2010). Intraoperative rest ECoG activity of the awake patient was acquired before the mapping session, re-referenced to the common average and transformed into the frequency domain using an autoregressive model for each electrode contact. The software then created a statistical model of baseline brain activity fitting a gaussian mixture model to the distribution of spectral estimates (Schalk et al., 2008a, b). During the mapping session, the patient received a visual and auditory cue in each trial to extend his phantom hand for 4 s, 2 s of finger or wrist extension, 2 s of holding finger or wrist extension, followed by a 4-s relaxation period. The patient was instructed to parallel this cycle by imagining moving his missing hand (phantom hand movement). The task-related ECoG activity was again re-referenced to the common average and transformed into the frequency domain using an autoregressive model for each electrode contact. The software detected significant task-related deviations from baseline by determining the negative log likelihood that the spectra at a specific location differed from the spectral distribution during the baseline period. The z-scores between the distributions of negative log likelihood values for the task and the interleaved rest periods were calculated during the trial to provide one continuously updated z-score for each electrode location and task. These values were shown on a topographical display for each task as a circle at each electrode location whose diameter was proportional to the absolute z-score at that location, thus enabling real-time assessment of cortical changes (Schalk et al., 2008a, b). Following five trials of each condition, the electrode grid position was refined under neuronavigational control. This entailed using preoperative magnetic resonance images to adjust the location of the strongest task-related ECoG activity to the center of the electrode grid (see **Figure 2**). This procedure was repeated several times to obtain an optimal orientation of the implant in the epidural space. It is worth mentioning that the whole mapping procedure took less than 15 min. Once implantation had been completed, the electrode grid was secured on the dura and externalized with percutaneous extensions connecting to the external component of the BCI set-up.

### IMPLANTED NEURAL INTERFACE

The epidurally implanted 8 × 8 electrode array consisted of platinum contacts with 4 mm contact diameter (2.3 mm exposed), and 5 mm center-to-center distance (Ad-Tech Medical Instrument Corp., Racine, WI, USA), covering parts of the left somatosensory, primary motor, and premotor cortex (see **Figure 4B**). This interface allowed for bi-directional exploration of cortical physiology in this patient by virtue of its ability to record brain activity related to phantom hand movements and to map cortical stimulation-induced sensations in the phantom hand. These physiological measures were then related to the patient’s cortical anatomy after co-registration of the electrode contacts to the three-dimensional reconstruction of the patient’s brain magnetic resonance image.

Following a 2 week evaluation period, the array was removed and replaced by permanent electrode leads for chronic application (Resume II, Medtronic, Minneapolis, MN, USA) at the sites where stimulation provided optimal pain control. As the described study was finished after the evaluation period, the pain treatment is not part of the present report.

### BRAIN–COMPUTER INTERFACE

**FIGURE 3:**
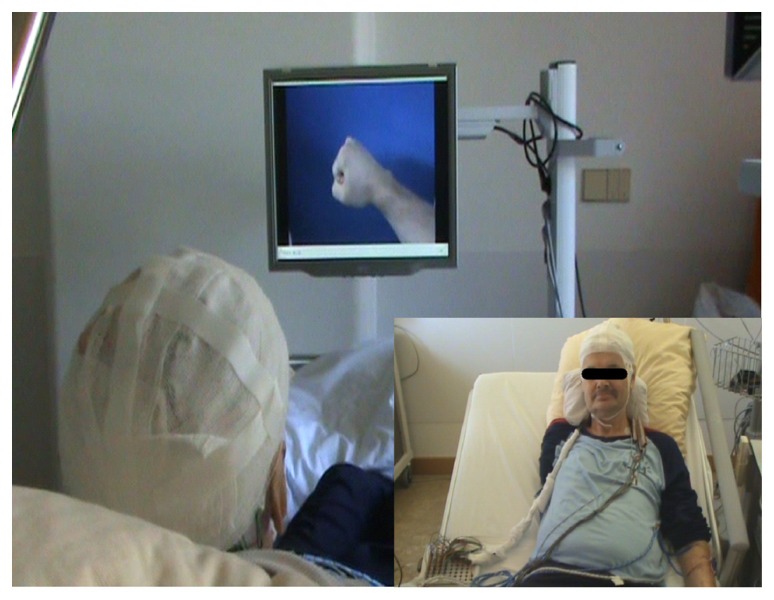
**FIGURE 3. Closed-loop brain–computer interface set-up.** The implanted epidural neural interface for electrocorticographic (ECoG) recordings is externalized with percutaneous extensions connecting to the external components (see small inserted picture). The external components consist of a recording and processing unit and a feedback unit. Recording of ECoG signals is performed with a monopolar amplifier (BrainProducts, Munich, Germany) with a high-pass filter at 0.15 Hz and a sampling rate of 1000 Hz. Online processing of brain signals is performed with a BCI 2000 framework (Schalk et al., 2004) extended with custom-built features to control a video player via UDP. Feedback is provided according to phantom movement-related brain activity by a video player displaying a virtual hand prosthesis from the patient’s perspective (Minimal Video Player, Phonon API).

The integrated system consisted of an internal component, the implanted epidural neural interface for ECoG recordings, externalized with percutaneous extensions connecting to the external components. These external components consisted of a recording and processing unit and a feedback unit. Recording of ECoG signals was performed with a monopolar amplifier (BrainProducts, Munich, Germany) with a high-pass filter at 0.15 Hz and a sampling rate of 1000 Hz. Online processing of brain signals was performed with a BCI2000 framework (Schalk et al., 2004) equipped with custom-built features to control a video player via a unified development platform (UDP). Feedback was provided according to phantom movement-related brain activity and was relayed by a video player that showed the virtual hand prosthesis (see **Figure 3**) from the patient’s perspective (Minimal Video Player, Phonon API).

The patient performed seven sessions, each of them on a separate day, with a total of more than 700 trials. In each session, classification of phantom hand movement versus rest was explored offline. From the third session onward, brain signals were also classified online and feedback of electrocorticographic activity was provided for real-time control of the virtual hand prosthesis. In each trial, the patient received a visual and an auditory cue to extend his phantom hand for 4 s (2 s of extension of all fingers or of wrist extension, 2 s of holding finger or wrist extension) followed by a 4-s relaxation period. The patient was instructed to parallel this cycle with his phantom hand. During the online sessions, feedback was provided in accordance with phantom movement-related brain activity by the virtual hand prosthesis which performed a hand opening motion according to the given instructions from the patient’s perspective while he was observing his hand. During the relaxation period the virtual hand was closing again. A short break was taken after every run (i.e., 10 trials).

### CLOSED-LOOP SET-UP

Prior to the five feedback sessions, two screening sessions were performed to select channels and features for online control, estimating the spectral power of all electrode channels up to 200 Hz and computing *r*^2^ scores for the discrimination between phantom hand movements and rest for each frequency bin and each channel. The three channels with the highest *r*^2 ^values for the frequency band of 130–145 Hz were chosen for feedback, because this was the topographically most circumscribed band for discrimination between phantom movement and rest. We considered the decoding of phantom hand movement from a small cortical area beneficial, as we intended to provide feedback of very specific cortical activations. Permutation tests with 10^5^repetitions confirmed that the *r*^2 ^values were significantly greater than 0. The selected channels and frequency band were kept constant throughout the training period.

During the feedback sessions, the spectral power of these three channels in the frequency band of 130–145 Hz was computed every 40 ms from a data buffer of length 500 ms. This was performed using an autoregressive model (Burg algorithm) with a model of the order of 16 (McFarland and Wolpaw, 2008b). These power values were used as input for an adaptive linear classifier, resulting in 25 classifier outputs per second. Five consecutive classifier outputs (200 ms) of the same feature were necessary to switch from one feedback condition to the other (e.g., to initiate or to terminate the opening of the virtual hand prosthesis).

### PERFORMANCE EVALUATION

To assess the patient’s ability to modulate his brain activity in accordance with the feedback task, i.e., his performance in gaining control of the virtual hand prosthesis, we calculated the average prosthesis movement time divided by the total feedback duration phase. We also measured a baseline condition to monitor natural perturbations of brain activity, which, while not related to the task, could have distorted the online control performance during the feedback task. This baseline condition consisted of several ECoG recordings throughout the 2-week study period; during recording the patient was at rest with his eyes open. All in all, about 30 min of this spontaneous baseline ECoG activity were recorded for offline analysis, segmented into trials of the same structure and processed in the same manner as the feedback sessions. For statistical analysis, the distribution of performance values per run in each feedback session was compared with the distribution of performance values for the baseline data (Wilcoxon rank-sum test).

## RESULTS

**FIGURE 4:**
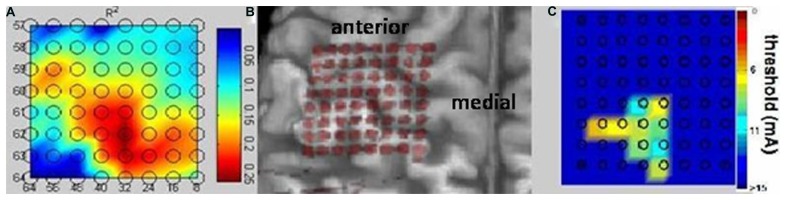
**Bidirectional epidural interface.** Projection of the implanted 64 contact electrode grid on a three-dimensional reconstruction of the patient’s MRI cortical anatomy revealing the correct intraoperative positioning of the epidural implant without direct visualization of the cortical surface during surgery **(B).** Physiological mapping confirms the implantation on the area of the sensorimotor cortex related to phantom hand movements and sensations and proves the feasibility of recording electrocorticography signals and mapping the cortical surface without direct contact with brain tissue. Both the high-gamma frequency power **(A)** and the cortical stimulation **(C)** revealed mutually overlapping representations and indicated preserved somatotopic organization of the sensorimotor cortex despite long-term amputation.

The approach implemented here allowed for sufficient positioning of the epidural implant on the area of the sensorimotor cortex related to phantom hand movements and proved feasible and safe for recording ECoG signals without direct contact to brain tissue in an upper-limb amputee (**Figure 4B**).

The high-gamma frequency power (**Figure 4A**) and the cortical stimulation (**Figure 4C**) both revealed mutually overlapping representations and indicated preserved somatotopic organization of the sensorimotor cortex despite long-term amputation without prosthesis use and despite accompanying intractable phantom limb pain. Sensations of the phantom hand could be elicited with high spatial resolution by epidural stimulation, revealing preserved sensory connectivity to the somatosensory hand knob area (**Figure 4C**). Interestingly enough, electrodes showing the highest *r*^2^ values in the high gamma band for phantom hand movement projected to the very same area, indicating preserved but posteriorly shifted cortical hand representation for motor control (**Figure 4A**).

The patient was able to participate in all five feedback sessions independently without requiring any assistance, while self-monitoring the translation of his phantom hand movement into the action of a virtual hand prosthesis. The epidural implant reliably detected electrocorticographic brain activity from the epidural space for providing real-time feedback of phantom movement-related brain activity. The ECoG signals, detected by the same three adjacent contacts with a center-to-center distance of only 5 mm projecting onto the somatosensory hand knob, were sufficient for consistent neurofeedback throughout the whole training period.

**FIGURE 5:**
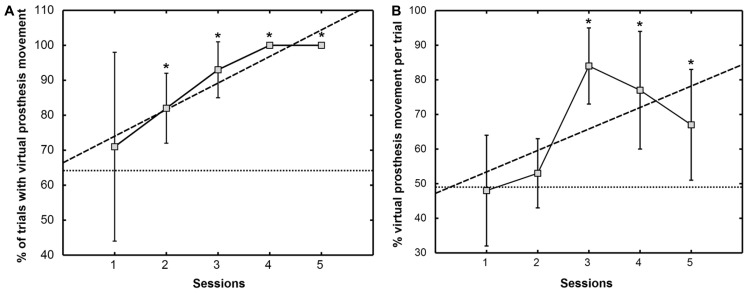
**Percentage of trials with virtual prosthesis movement **(A)** and percentage of average movement time of the virtual prosthesis divided by the total feedback duration phase **(B)**.** The mean ± SD of the performance measure per session is indicated by solid lines. Dashed lines show the trend line determined by linear least square regression. The mean of the baseline data is given as a dotted line. An asterisk (*) marks sessions where the median of the performance measure differs significantly (*p* <0.05) from the median of the baseline value [modified from Walter et al. (2012), presented at the 12th International Conference on BioInformatics and BioEngineering, Larnaca, Cyprus].

From the third feedback session onward, the patient was able to initiate prosthesis movement in more than 95% of the trials, indicating preserved task-contingent brain activity during phantom movement (**Figure 5A**). His performance in closed-loop control of the virtual prosthesis, i.e., online movement control during the feedback task, was significantly higher than in the baseline condition (49.1 ± 10.6%) from the third feedback session on (**Figure 5B**).

## DISCUSSION

While BCI/BMI for prosthetic control have been explored in able-bodied individuals, similar experience in amputees is still somewhat limited.

The Berlin BCI group used EEG recordings to investigate the readiness potential and event-related desynchronization (ERD) of eight upper-limb amputees during real finger movement of the intact side and during phantom finger movements (Kunzmann et al., 2004; Blankertz et al., 2006). They observed less distinct ERD during phantom movement, a decrease of detection accuracy proportional to the time elapsed since the limb was lost, and offline classifications for “right vs. left” of between 60 and 78%. This limited accuracy is very probably related to the low signal-to-noise-ratio due to signal attenuation caused by the skull inherent to non-invasive BCI approaches using EEG (Leuthardt et al., 2009).

The Osaka ECoG group were the first to apply an implantable BCI approach to an amputee (Yanagisawa et al., 2012). Within the framework of a pain treatment protocol similar to the one conducted in this study, the Osaka group implanted a subdural grid on the sensorimotor cortex of a patient 3.3 years after transhumeral amputation, gaining offline classification accuracies of 66.3% for grasp vs. elbow and 89.2% for move vs. rest.

However, some essential questions concerning the clinical viability of implantable BCI approaches for amputees remained unanswered. For instance, is a high-level upper-limb amputee capable of generating contingent cortical activity with intuitive motor commands for gaining online-control of a hand prosthesis? Are these signals robustly detectable over several days without the necessity of re-calibration or periodical updating of the neural decoding? Are less invasive approaches for BCI control, such as epidural implants and a few decoding channels, feasible in this group of patients? Does cortical reorganization following long-term amputation compromise the detection of natural movement-related brain signals?

We therefore introduced a methodology to implant a cortical interface without direct exposure of the brain surface in an upper-limb amputee, and implemented a closed-loop set-up based on an *epidural* implant that provided real-time feedback of motor-related electrocorticographic brain activity to operate a virtual hand prosthesis following *long-term* high-level limb deficiency.

In accordance with earlier reports on able-bodied individuals (Leuthardt et al., 2006), we could show now for the first time in an amputee that epidurally recorded signals were appropriate for reliable BCI feedback, thereby further increasing the safety of such applications with intracranial recordings. Moreover, the same three adjacent electrode contacts were sufficient for prosthetic control throughout the evaluation period, indicating that smaller electrode grids with fewer contacts might suffice for future applications of this technique. These results tally with recent observations in epilepsy patients who had been subjected to a BCI visual speller with a single subdural contact (Zhang et al., 2013). Thus, our findings emphasize the feasibility of less invasive and more straightforward approaches, with only a small number electrode contacts in the epidural space for applications of assistive ECoG BCI.

In addition, throughout the feedback sessions, no adjustments of the parameterization between the recorded brain signals and the BCI control algorithms were necessary. These findings are in accordance with a report of multiple-day ECoG BCI control with fixed parameters in an able-bodied patient for seizure localization (Blakely et al., 2009). Similarly, we performed an initial screening and feature selection followed by a robust and stable-state control of the interface from the third online session onward. This is an important requirement for future real-life applications of such tools for daily use, without the necessity of periodical software adaptations by specialists at respective institutions. Interestingly enough, the patient who achieved this performance had no experience of prosthetic use beforehand.

Despite long-term amputation (26 years ago) and despite intractable phantom limb pain throughout this period, channels projecting onto the somatosensory hand knob of the affected hemisphere provided sufficient information about the phantom hand movement to allow robust and stable prosthesis control, indicating that the somatotopic organization of the corresponding brain has been at least partially preserved. This demonstrated that even patients with long-term deficiencies may be suitable recipients of implantable BCI devices that address prosthetic control.

Electrocorticography studies in patients without motor deficits have shown specific movement-related spectral changes, i.e., decreases in the low-frequency band (8–32 Hz) with a larger cortical distribution and increases in the high-frequency band (76–100 Hz) with a more focused projection onto the sensorimotor cortex (Miller et al., 2007). In addition, ECoG recordings in these patients allowed for both temporally and spatially precise and robust online detection of movement imagery/intention in the absence of real movement (Miller et al., 2010).

We consequently detected a circumscribed increase of cortical activity in the high gamma band during phantom hand movement. Interestingly enough, these electrodes projected to the somatosensory hand knob area, the very same area in which cortical stimulation elicited sensations of the phantom hand. This observation indicated that the same cortical area with sensory connectivity to the missing hand presented the posteriorly shifted cortical hand representation for motor control. These findings are in accordance with previous observations in epileptic patients who underwent implantation of subdural electrode grids in the sensorimotor region (Nii et al., 1996; Haseeb et al., 2007). These studies revealed hand motor responses following electrical stimulation not only in the precentral gyrus but also in the postcentral gyrus indicating a marked variability in the location of the human cortical hand area (Nii et al., 1996; Haseeb et al., 2007). This may well have implications for future designs and configurations of bi-directional cortical interfaces for amputees.

The BCI set-up enabled us to make an update of the control signal as often as every 40 ms. However, our algorithm did not provide visual feedback until five consecutive 40 ms epochs had been classified consistently, thus avoiding a noisy control signal or false positive feedback. This trade-off between immediacy and contingency was sufficient both for robust BCI control and for the patient to comprehend that there is a direct link between his phantom movement and the virtual prosthesis movement. This is in agreement with earlier psychophysical studies in which participants who were repeatedly exposed to an artificially introduced 250 ms delay between voluntary actions and sensory consequences perceptually combined their voluntary actions with the sensory consequences and perceived that the delay was shortened by approximately 100 ms (Haggard et al., 2002).

Due to the lack of cortical implants allowing for wireless BCI control, we designed an interface by connecting the intracranial implant to an external online processing framework for recording and BCI control. To this end, extension leads were externalized through the skin and thus limited the possible duration of this set-up. Wireless devices capable of fast and reliable information transfer will be required for future clinical applications of this novel approach (Borton et al., 2013). This would make it possible to conduct long-term training periods of this paradigm in home-based environments, and hopefully result in better online control and higher dexterity to improve the patients’ quality of life.

The present findings indicate that cortical neural prosthesis might serve as a natural source of information to facilitate new control strategies by combining brain and peripheral nerve signals (Tombini et al., 2012) and to support more intuitive shared control strategies, e.g., arranged in a hierarchical manner with myoelectric signals, necessitating less attention, and consecutive fatigue (Cipriani et al., 2008). Additionally, this cortical interface methodology allows for closing the loop by providing sensory feedback via direct stimulation of the central neural system.

In conclusion, epidural implants may constitute a powerful and safe alternative communication pathway between the brain and external devices for upper-limb amputees. Such implanted brain interfaces can support decoding movement intention of the missing hand in an intuitive way. This might provide additional information on the standard myoelectric and neural biosignals applied in upper-limb prosthesis, thereby facilitating the integrated use of different signal sources for more specific control of multi-functional devices in clinical use.

## Conflict of Interest Statement

The authors declare that the research was conducted in the absence of any commercial or financial relationships that could be construed as a potential conflict of interest.
